# Antipsychotic-induced hyperprolactinemia: Toxicologic mechanism and the increased breast cancer risk

**DOI:** 10.1016/j.toxrep.2025.101927

**Published:** 2025-02-01

**Authors:** Steven B. Bird

**Affiliations:** UMass Chan Medical School, 55 Lake Avenue North, Worcester, MA 01545, USA

**Keywords:** Antipsychotic, Bipolar disorder, Breast cancer, Neuroleptic, Prolactin, Schizophrenia

## Abstract

Antipsychotic drugs are effective at improving both the positive and negative symptoms of schizophrenia as well as the manic phase of bipolar disorder. Whether an antipsychotic is termed typical or atypical is related to the xenobiotic’s propensity to cause extrapyramidal side effects. However, with a few exceptions, drugs of both classes of antipsychotics are known to cause hyperprolactinemia. As many breast cancers are responsive to prolactin concentrations, the persistent increase in prolactin of the antipsychotics has implications for public health and carcinogenesis. The objective of this study was to review the extant literature on hyperprolactinemia due to antipsychotics, and to determine the risk imposed by those drugs on human breast cancer. A summary risk of breast cancer with use of any antipsychotic was found to be 1.19 (95 % confidence interval 1.10–1.30). When limiting usage of antipsychotics to 5 or more years, the summary risk increased to 1.26 (95 % confidence interval 1.12–1.43). And when limited to those studies who evaluated only those medications with the greatest increase in prolactin, the risk increased to 1.59 (95 % confidence interval 1.37–1.85). Given this increased risk of breast cancer, stronger warnings about this increased risk are warranted, and regular monitoring of prolactin levels and breast cancer screening should be part of the management plan for these patients.

## Highlights

• Antipsychotics cause a persist increase in prolactin in humans.
• Both typical and atypical antipsychotics can cause the increase in prolactin.
• Use of any antipsychotic was found to increase the risk of breast cancer by 19 %.
• Longer use of antipsychotics had an increased risk of breast cancer of 26 %.
• Drugs with the most effect on prolactin had an increased risk of 59 %.

## 1 Introduction

Antipsychotic drugs, sometimes termed neuroleptics, are primarily used to treat schizophrenia, bipolar disorder, as well as acute agitated behavior. Antipsychotics are a structurally diverse group of heterocyclic compounds with numerous oral and injectable formulations available for clinical use worldwide. Although traditionally classified by their molecular structure, antipsychotics are more ideally classified by pharmacological profile. Each drug has a unique receptor-binding profile which can be used to predict adverse effects in both therapeutic use and overdose.

In addition to receptor-binding characteristics, antipsychotics are also commonly classified as “typical” or “atypical”, or as “first generation” or “second generation.” Such classifications are referable to their propensity for causing extrapyramidal signs (EPS). Typical antipsychotics are those which can produce EPS, while atypical antipsychotics are newer drugs that have a lower extrapyramidal side effect profile.[5]

Another approach to categorizing antipsychotics is based on their tendency to increase prolactin concentrations in users of the drug. Prolactin is a hormone primarily produced and secreted into the bloodstream by the anterior lobe of the pituitary. Prolactin is crucial for the cellular expansion as well as differentiation of mammary gland tissue.[15] In mice, deletion of the prolactin gene or its receptors prevents lactation.[50] The physiological activities of prolactin are largely breast expansion during pregnancy and the initiation and maintenance of breastfeeding.[7] Although prolactin is commonly regarded as a hormone produced by the pituitary gland, its synthesis and secretion are not confined solely to this region; extrapituitary locations, including the mammary glands, can also produce prolactin.[17] Consequently, prolactin is delivered to the breast via both systemic circulation (from the pituitary) and locally (from breast tissue).

The regulation of prolactin production and secretion varies between pituitary and extrapituitary locations.[17] In typical physiological circumstances, the synthesis and secretion of prolactin by the anterior pituitary is continuously suppressed by the hypothalamus, through various prolactin inhibitory factors - with dopamine being the most significant among them.[38], [24] Stimulators of prolactin secretion include pregnancy [23], breast feeding [68], physical and psychological stress [39], exercise [57], and hypothyroidism [8]. The antagonism of dopamine D2 receptors by antipsychotic medications decreases the inhibitory effect of dopamine on prolactin secretion; this results in a disinhibition of prolactin release. The extent of dopamine D2 receptor antagonism correlates positively with the elevation of prolactin levels.[52] Although pituitary prolactin is predominantly governed by dopamine, extrapituitary prolactin exhibits insensitivity to dopamine.[4], [15] This explains the ineffectiveness of dopamine agonists in influencing prolactin-dependent cancer in humans [37], while in people with a pituitary prolactinoma, agonists such as bromocriptine successfully normalize pituitary prolactin concentrations and reduce tumor size.[21]

The D2 receptor antagonism of antipsychotics leads to increased prolactin secretion and a resulting hyperprolactinemia (generally defined as a serum prolactin concentrations greater than 20 ng/ml in men and 25 ng/ml in women)[52]. All of the antipsychotics can increase prolactin concentrations to some degree. However, significant differences exist among the class of drugs with regards to the degree of prolactin elevation. Hyperprolactinemia is most frequently observed with use of first-generation antipsychotics and the second-generation antipsychotics risperidone, paliperidone, and amisulpride.[6] Clozapine and other second-generation antipsychotics such as aripiprazole and quetiapine have the smallest effect on prolactin secretion[52] (Table 1). Furthermore, some antipsychotics also exhibit partial dopamine agonism (such as aripiprazole), which can lead to decreased prolactin levels.[75] The potential for aripiprazole to decrease the occurrence of breast cancer has not been investigated, but aripiprazole has been studied as an adjunct to chemotherapy in cancer cell lines (independent of its affect on prolactin) [3]. Antipsychotic-induced hyperprolactinemia generally occurs soon following the initiation of treatment (as early as within a week)[63] often leading to sexual side effects such as decreased libido[62], amenorrhea[66], and galactorrhea[22].However, long-term hyperprolactinemia that is asymptomatic is also prevalent, raising concerns regarding its long-term implications.[27]

**Table 1:** Antipsychotic drugs as well as their generation and propensity to increase prolactin. Data for the propensity to increase prolactin is taken from numerous sources, including [14], [19], [27], [28], [29], [40], [44], [52], [59], [6], [67]. Differences in methodology, dose of antipsychotic, and concomitant medication use makes the propensity designation shown to be estimates.

Generic Name	Generation	Prolactin Elevation
Amisulpride	2nd	3 +
Aripiprazole	2nd	0
Asenapine	2nd	1 +
Brexpiprazole	2nd	1 +
Cariprazine	2nd	0
Chlorpromazine	1st	2 +
Clozapine	2nd	1 +
Fluphenazine	1st	2 +
Haloperidol	1st	3 +
Iloperidone	2nd	2 +
Loxapine	1st	2 +
Lurasidone	2nd	2 +
Olanzapine	2nd	2 +
Paliperidone	2nd	3 +
Perphenazine	1st	2 +
Pimavanserin	2nd	0
Pimozide	1st	2 +
Quetiapine	2nd	1 +
Risperidone	2nd	3 +
Sertindole	2nd	1 +
Thiothixine	1st	2 +
Thioridazine	1st	2 +
Trifluoperazine	1st	2 +
Ziprasidone	2nd	1 +
Zotepine	2nd	1 +

Experimental and epidemiological evidence increasingly implicate a role for prolactin in breast cancer.[74], [1] Hyperprolactinemia-inducing antipsychotics have been shown to increase breast cancer risk by activating the JAK-STAT5 pathway in precancerous lesions in mice.[31] This activation leads to suppression of apoptosis, thereby promoting the progression of precancerous cells to cancerous ones. Another study by Dong *et al.* investigated the effects of olanzapine in rats.[16] Olanzapine induced significant mammary gland development and histopathological hyperplasia, which was dose- and time-dependent and was associated with increased prolactin receptor expression in the mammary gland.

Similar to the development of usual mammary gland tissue, carcinogenesis of breast tissue is largely a hormonally-dependent process.[48] Consequently, female reproductive hormones such as estrogen and progesterone, along with the hormone prolactin, may significantly influence breast cancer risk and tumor development. This observation has prompted inquiries regarding the potential association between antipsychotic-induced hyperprolactinemia and breast cancer.

In 2023 and 2024, 5 studies were published examining the association of antipsychotic use and breast cancer. However, given the close approximation of their publication, none of the more recent studies were able to consider all published data. The goal of this investigation was to perform an updated review the literature including these new studies that examined antipsychotic sue and breast cancer, and to generate a summary statistic for a possible association.

## 2 Material and methods

The Preferred Reporting Items for Systematic Reviews and Meta-Analyses (PRISMA) checklist was utilized in conducting this review.[51] A search of English-language articles published in peer-reviewed journals in PubMed from 1950 until November 1, 2024 was undertaken. The specific search terms and combinations of keywords and text words descended from previously published reviews and a general review of the topic.[35], [41].

**Fig. 1:**
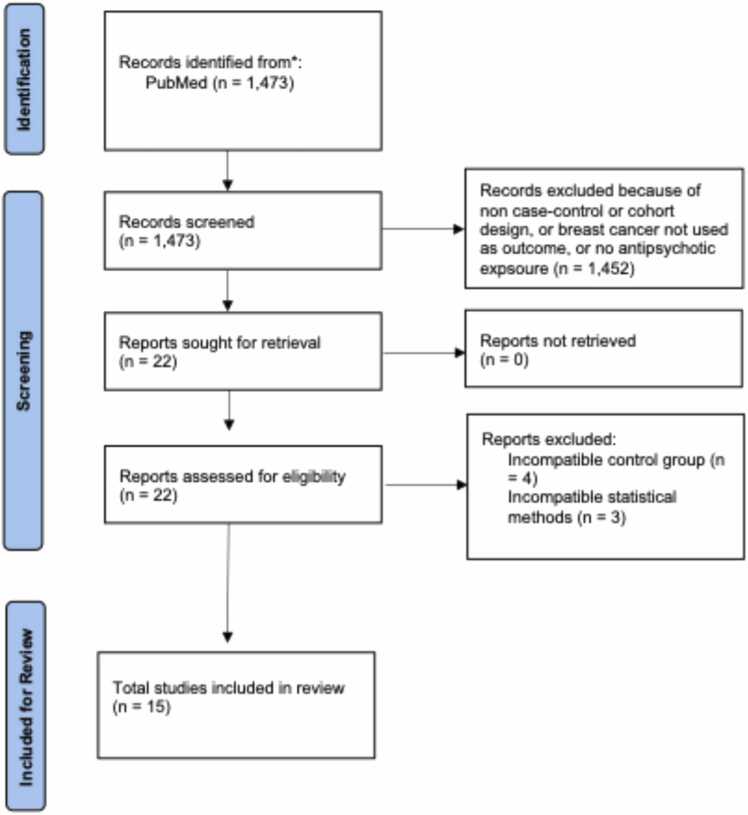
PRISMA flow diagram.

The specific search terms were the following: All fields: [antipsychotic OR neuroleptic OR tranquilizer OR "tranquillizing agent" OR psychotropic OR "dopamine receptor blocker" OR "dopamine antagonist" OR "dopamine receptor blocking agent" OR "D2 receptor blocker" OR "D2 antagonist" OR "D2 receptor blocking agent" OR "typical antipsychotic" OR "atypical antipsychotic" OR "first generation antipsychotic" OR "second generation antipsychotic" OR sertindole OR zotepine OR chlorpromazine OR droperidol OR fluphenazine OR haloperidol OR loxapine OR perphenazine OR pimozide OR prochlorperazine OR thiothixene OR thiotixene OR thioridazine OR trifluoperazine OR apiprazole OR asenapine OR clozapine OR iloperidone OR olanzapine OR paliperidone OR quetiapine OR risperidone OR ziprasidone OR amisulpride OR brexpiprazole OR cariprazine OR pimavanserin OR lurasidone] AND all fields: ["breast cancer" OR "breast tumor" OR "breast lesion" OR "breast neoplasm" OR "breast carcinoma" OR "breast malignancy" OR "mammary cancer" OR "mammary tumor" OR "mammary lesion" OR "mammary neoplasm" OR "mammary carcinoma" OR "mammary malignancy" OR "ductal carcinoma" OR "lobular carcinoma" OR "breast cancer risk" OR "breast cancer incidence" OR "mammogra* " OR "breast tumorigenesis" OR "breast carcinogenesis" OR "cancer of breast" OR "cancer of the breast" OR "malignant tumor of breast" OR "malignant tumor of breast"]. Results of the search are presented in Fig. 1.

## 3 Selection of studies – anti-psychotic use and breast cancer

Initially, titles and abstracts were evaluated. Then, the studies were selected for complete reading. Furthermore, the bibliography of each reviewed study was also reviewed in an effort to identify additional studies that may not have been identified with the MEDLINE search terms. Studies that contained data regarding the use of antipsychotics as well as quantitative data on breast cancer occurrence, the absolute number of patients, or risk ratios between the evaluated groups were included.

## 4 Data extraction and qualitative study assessment

The following data were extracted from each manuscript: author, publication date, study design, mean duration of follow-up, minimum required period of antipsychotic use to be deemed a user of the medication, as well as population characteristics such as number of patients, number of controls, and the type of antipsychotic drugs used in the study. The Newcastle Ottawa scale was used to assess quality of studies. Studies were then categorized based upon selection, comparability, and results. Scores used were > 8 were considered as high quality; score between 4 and 7 were considered as moderate quality; and a score < 3 were considered as low quality.[64]

For each study included in this analysis, the reported Odds Ratio, Hazard Ratio, or Relative Risk of developing breast cancer following exposure to antipsychotic medications, along with the 95 % confidence intervals for these measures, were extracted. The log risk measure and confidence intervals were used as the input for the "metagen" function from the R package "meta". The between-study variance was estimated using the DerSimonian-Laird method, and heterogeneity between studies was assessed using Cochran's Q test and the I^2 statistic. The studies were pooled using the inverse variance method, a random effects meta-analysis was performed, and the resulting risks and confidence intervals were reported.

## 5 Results

Upon initial PubMed search a total of 1473 articles were identified. A review of the titles and abstracts resulted in excluding 1451 articles for either employing something other than a cohort or case-control design, not using breast cancer as an outcome of interest, or not including antipsychotic medication use. Of the remaining 22 articles, 7 were excluded for a incompatible statistical methodology or incompatible/absent control groups.

Among the 7 articles excluded from analysis, the Azoulay 2011[2] article compared typical to atypical antipsychotics, and therefore had no control group. The article by Kanhouwa *et al.* Kanhouwa et al., [32] was not included because it was restricted only to in-patients who got anti-psychotics and had no control group. The study by Kelly et al. *Kelly* [33] was eliminated because they used 2 control groups: one group had cancer (but not breast cancer), and the other control group did not have any form of cancer. However, relative risks of breast cancer were not provided. The 2021 study by Maeshima [45] used data from the Japanese adverse event reporting system and included basic science mechanistic results. However, the authors did not provide any summary statistics for breast cancer occurrence. The 1987 article by Mortensen was not included in this analysis because they looked at a number of cancers (including breast), but provided no risk estimates for the occurrence of breast cancer.[49] The articles by Reutfors [55] and Tsai [70] were also not included because they had no control group; patients in those two studies were compared to users of other antipsychotics. Study characteristics of the articles included in this review are show in Table 2.

**Table 2:** Details of the 15 included studies.

Author	Year	# users of anti-psych	Mean Use of Med	Mean Follow-up (yrs)	Study Design	Anti-psychotic type	OR (95 % CI)	Study Location	Study Quality
Chou	2017	10727	NR	NR	retrospective cohort	all	1.94 (1.43–2.63)	Taiwan	high
Chu	2023		NR (at least 1 year)	NR	nested case-control	all	1.50 (1.11–2.01)	Hong Kong	high
Dalton	2006	15556	> 2 months	6.4	cohort	all	0.93 (0.74–1.17)	Denmark	high
George	2020	361	NR	7.8	cohort	all	1.06 (0.93–1.21)	US	high
Hippisley-Cox	2007	122	NR	NR	nested case-control	all	1.55 (1.08–2.23)	UK	high
Joo	2022	498970	1 year	5.2	nested case-control	2nd gen	1.08 (1.04–1.13)	Korea	high
Kato	2000	92	NR	7.3	prospective chort	all	2.0 (0.89–4.46)	US	moderate
Kern	2024	4256	at least 180 days	4	retrospective cohort	hi v low PRL	1.05 (0.68–1.78)	US	moderate
Li	2024	17104	NR	4.1	nested case-control	all	0.77 (0.62–0.95).	Taiwan	high
Pottegard	2018	52594	NR	NR	case-control	all	1.18 (1.06–1.32)	Denmark	high
Rahman	2022	312702	NR	4.75	cohort	all	1.35 (1.14–1.61)	US	high
Solmi	2024	132061	years	4	nested case-control	all	1.32 (1.13–1.53)	Sweden	high
Taipale	2021	30785	NR	6.5	nested case-control	all	PRL increasing 1.56 (1.27–1.92)	Finland	high
Wang	2002	52819	NR	NR	retrospective cohort	all	1.16 (1.07–1.26)	US	high
Yang	2024		NR	7.1	case-control	all	1.26 (1.20–1.32)	Korea	high
									
NR=not reported									

**Fig. 2:**
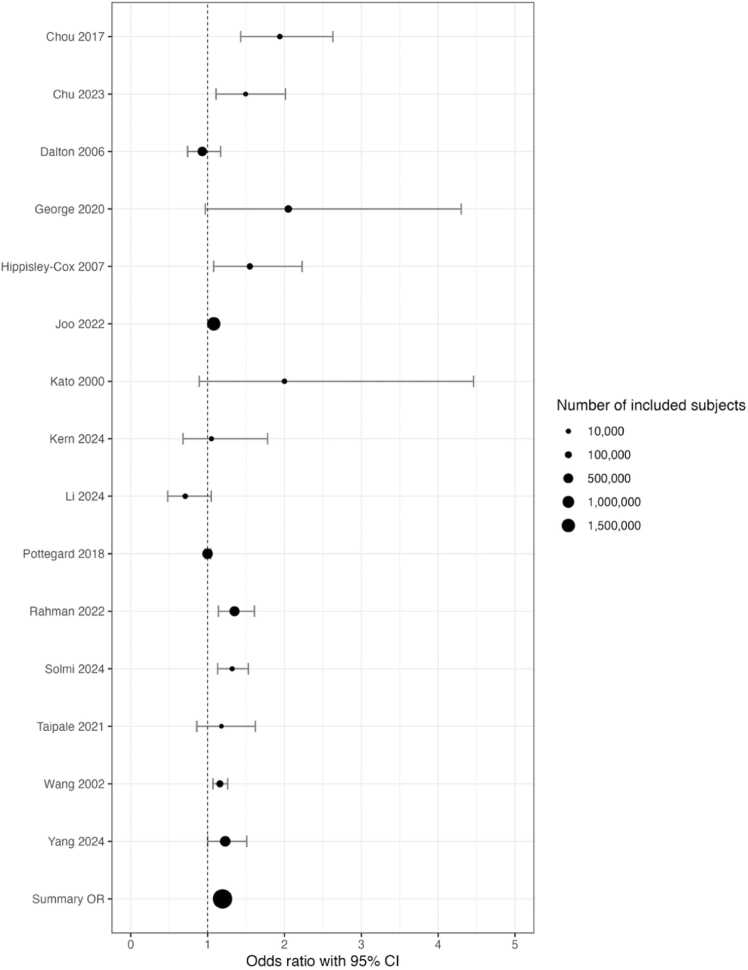
Risk of breast cancer with any antipsychotic use of any duration, from the 15 included studies.

From the 15 studies included in this review, the overall risk of breast cancer in users of any antipsychotic at any dose and for any duration was found to be 1.19 (95 % CI 1.10–1.30) (Fig. 2). However, cumulative daily dose or median daily dose of the antipsychotics was only rarely reported in the 15 included studies. There was statistically significant heterogeneity across all of the studies (Q = 67.71, p < .001, I2 = 79 %). For those studies that looked at long term antipsychotic use, there was also statistically significant heterogeneity (Q = 32.57, p < .001, I2 = 79 %).

For the few studies that specifically examined high-prolactin increasing medications, statistically significant heterogeneity was not observed (Q = 0.06, p = .80, I2 = 0 %).

It is important to consider the cumulative dose of a drug when evaluating its carcinogenic adverse effects because the risk of carcinogenesis often correlates with the total amount of exposure over time. This relationship is grounded in the principle that the cumulative dose can influence the extent of DNA damage and the subsequent risk of mutation and cancer development.

For instance, the article by Chou demonstrated that the dose-effect relationships of carcinogens conform to the median-effect principle, which incorporates cumulative dose as a critical factor in assessing carcinogenic risk.[9] Additionally, the article by Clewell et al. [13] discusses the non-linear dose-response relationships for both genotoxic and non-genotoxic carcinogens, indicating that cumulative exposure can lead to different biological mechanisms of action, which are crucial for accurate risk assessment.[13]

**Fig. 3:**
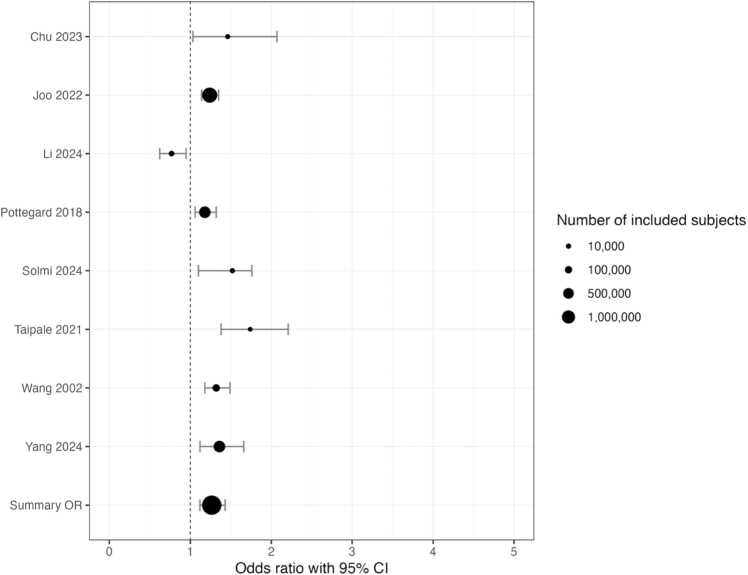
Risk of breast cancer in those studies that examined antipsychotic use for 4 or more years.

To account for cumulative exposure to antipsychotics, use for at least 4 years was also investigated. However, the 15 studies did not routinely report use duration or the mean duration of use. When looking at use for 4 or more years when those data were explicitly stated in a manuscript, the risk of breast cancer increased to1.26 (95 % CI 1.12–1.43 (Fig. 3).

**Fig. 4:**
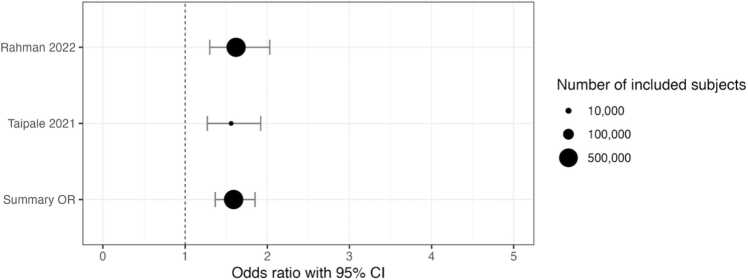
Breast cancer risk from the 2 studies that specifically examined high-prolactin increasing antipsychotics.

Because hyperprolactinemia is thought to be an independent risk factor for breast cancer, the use of high-prolactin secreting anti-psychotics was also examined. Although only reported by 2 of the studies, when limiting the evaluation to only those antipsychotics, the risk of breast cancer increased further to 1.59 (95 % CI 1.37–1.85) (Fig. 4).

## 6 Discussion

In this review, summary statistics of breast cancer risk demonstrated an overall 19 % increased risk of breast cancer in women who take antipsychotics. This increased risk was evident when combining all antipsychotics and when looking specifically at those drugs which increase prolactin to the greatest degree. While few studies examined or reported any sort of dose-response, a summary of those studies revealed a 26 % increased risk of breast cancer with antipsychotic use for 4 or more years.

Studies have found that the prolactin receptor is overexpressed in breast cancer compared to non-cancer tissues.[43]. Furthermore, prolactin helps to promote the proliferation, survival, and chemotaxic ability of breast cancer cells.[46]. Hyperprolactinemia is also associated with an elevated risk of developing metastatic breast cancer.[12] In transgenic mice with overexpression of prolactin develop ER+ (estrogen receptor positive) or ER− (estrogen receptor negative) mammary carcinomas early in life.[58]. Numerous studies have found that higher blood concentrations of prolactin lead to an increased risk of ER+ breast cancers in both postmenopausal [72] and premenopausal women.[18]. Furthermore, the Nurses’ Health Study found that prolactin concentrations predicted breast cancer risk.[71] Lastly, in what could represent an additional method of carcinogenesis, studies have found an association to prolactin concentrations and breast density, which is a recognized risk factor for breast cancer. Studies have also linked prolactin to breast density on mammogram [25], a known and recognized independent risk factor for breast cancer.[30]

Antipsychotic classification as fist generation or second generation based on their initial clinical use market introduction and potential to induce extrapyramidal adverse effects. This somewhat arbitrary classification easily can lead to misunderstanding about a drug’s likelihood to increase blood prolactin concentrations.[54] For example, many typical or first-generation antipsychotics exhibits the greatest propensity to elevate prolactin concentrations, while the atypical or second-generation drugs risperidone and paliperidone also exhibits similar effects on prolactin.[40], [44]. Further adding to confusion on this topic, different studies utilize different protocols for dosing and the timing of prolactin measurements. Thus, a drug could have little or no effect on prolactin secretion at low doses but could demonstrate significant increases in prolactin at higher doses. For instance, even at relatively low doses, risperidone can increase prolactin concentrations, and this effect becomes more pronounced at higher doses (positive dose-response).[73], whereas clozapine has been shown to have minimal effects on prolactin levels at therapeutic doses, but at very high doses it can cause moderate increases in prolactin concentrations.[47]

The potential for increased breast cancer or more aggressive breast cancer with antipsychotic use has been recognized by the Food and Drug Adminstration (FDA). The FDA has issued warnings about antipsychotic medication use in patients with a history of breast cancer, primarily due to the potential for these drugs to elevate serum prolactin levels. In warning label for Jannsen Pharmaceuticals Risperdal-branded risperidone includes the following: “As with other drugs that antagonize dopamine D2 receptors, RISPERDAL® elevates prolactin levels and the elevation persists during chronic administration. RISPERDAL® is associated with higher levels of prolactin elevation than other antipsychotic agents.”[56]

Elevated prolactin levels have been implicated in the progression of breast cancer, as prolactin can promote the growth of breast cancer cells.[53] Specifically, the FDA notes that antipsychotic drugs, can increase prolactin levels, which may be of concern in patients already diagnoses with breast cancer.

These results are consistent with results from previous studies. The Taipale case-control study from Finland found that the risk of breast cancer increased by 56 % in women with schizophrenia who were treated with antipsychotics for five or more years compared to those who did not use antipsychotics.[65] These results are also consistent with the study by Rahman, who examined more than 500,000 women in the U.S. and found that antipsychotics which cause hyperprolactinemia were associated with an increase in breast cancer.[54] In a meta-analysis of antipsychotic use and breast cancer by Leung *et al.*, they found a roughly 30 % increased risk of breast cancer with any antipsychotic use, regardless of the drug’s effects on prolactin blood concentration.[41]. Since publication of the above three cited studies, several other authors have likewise found an increased risk of breast cancer with antipsychotic use.

Chu *et al.* investigated the occurrence of breast cancer in women with bipolar disorder or schizophrenia who were treated with antipsychotics. Chu [10] They found both an increased risk of breast cancer with both first generation and second generation antipsychotic use, as well as an increased risk with 4 or more years drug use. Although the results for bipolar and schizophrenia were reported separately, when the breast cancer risk for the two disorders are combined, the risk with antipsychotic use was 1.46 (95 % CI 1.03–2.07). A similar study design by Li *et al.* from Taiwan investigated breast cancer occurrence in women treated with antipsychotics for bipolar or major depression.[42]. They compared the incidence of breast cancer with antipsychotic use by cumulative defined daily dose (cDDD). They used less than 30 cDDD as the referent group and compared that group to those who used 30–170 cDDD, 180–364 cDDD, and > 365 cDDD. Using these exposure groups, neither first generation nor second generation antipsychotic use were associated with an increased incidence of breast cancer. One significant limitation to the Li study, however, is obviously the duration of use: it would not be expected that 180 days of use of a drug would increase the risk of cancer. If that were so, we likely would have known or expected about an association of antipsychotic use and breast cancer long ago.

Another publication from 2024 by Solmi *et al.* Solmi [61] was a nested case-control study from Swedish registries. They examined the records of more than 130,000 women (of whom 1642 (1.24 %) developed breast cancer) with a mean age of 63.3 years and compared them with 8173 matched controls. The authors identified an odds of breast cancer in women with use of prolactin-increasing antipsychotics for 1–4 years (OR 1.20; 95 % confidence interval 1.03–1.41), and for ≥ 5 years (OR 1.47; 95 % confidence interval 1.26–1.71). They did not detect an increased odds of breast cancer with use of prolactin-sparing antipsychotics of either 1–4 years (OR 1.17; 95 % confidence interval 0.98–1.40) or ≥ 5 years (OR 0.99; 95 % confidence interval 0.78–1.26).

While the summary statistic of studies reviewed here show a positive association of antipsychotic use and breast cancer, not every study that has examined antipsychotic use and breast cancer has found such an association. For instance, the study by Kern et al. examined the occurrence of breast cancer in women who used high prolactin-increasing antipsychotics to those who used a low-prolactin increasing medication, with a minimum use period of 180 day.[34] This industry-sponsored study did not detect a statistically-significant increase in breast cancer among the high-prolactin increasing group.

Overall, women with schizophrenia have elevated rates of breast cancer risk factors, such as obesity, diabetes, and smoking. Additionally, they have lower rates of ever being pregnant and of breastfeeding compared to the general population, both of which also increase the risk of beast cancer.[60]. Therefore, studying the contribution of antipsychotic use to the occurrence of breast cancer in women with schizophrenia requires controlling for those factors or selecting appropriate comparators.

Male breast cancer accounts for just 0.6 % of all breast cancers. Giordano [20]. Therefore, due to its rare occurrence in men, the association between antipsychotic use and male breast cancer has not been investigated. Were an increased risk of breast cancer identified in men who used antipyschotics, that would lend strong evidence of an association. Given male breast cancer rarity, it is unlikely that such evidence will be forthcoming.

As with all studies, the current study has several limitations. First, the literature review and included data are all from observational studies. For obvious reasons, no prospective or randomized studies of antipsychotic use and breast cancer exist. Therefore, there are likely to be some unidentified or unmeasured confounders within the studies. Second, the studies included in this review had heterogeneous methodologies, such as duration of exposure to the antipsychotics, duration of follow-up, and differences in the doses used of each drug. However, these different methodologies would bias the result towards the null, which strengthens further the finding of an increased risk of breast cancer here. Furthermore, variable inherent breast cancer risk in women of different ethnicities[36] would tend to obscure risks for those with an increased baseline risk of breast cancer. Similarly, the risk of breast cancer in premenopausal and postmenopausal women, as well as the risk based upon ER and PR positivity are also worthy of further examination.

It is also important to recognize that while the findings presented here are clinically relevant, undertreating schizophrenia has significant short- and long-term implications in patients both with and without cancer. Undertreating schizophrenia with antipsychotics after the first onset of psychotic symptoms are associated with more severe symptomatology at both hospital admission and upon discharge, including more severe negative symptoms and a more severe reality distortion syndrome.[26] Additionally, longer durations of untreated psychosis are linked to poorer functional and symptomatic long-term outcomes. For instance, each increment in the duration of untreated psychosis is associated with a decrease in global functioning and an increase in positive symptom scores.[11] Moreover, untreated or undertreated schizophrenia increases the risk of relapse and rehospitalization. Patients who discontinue antipsychotic treatment or receive inadequate treatment have a higher risk of relapse and rehospitalization compared to those who receive continuous antipsychotic treatment.[69]

## 7 Conclusion

There is strong biological plausibility for an increased risk of breast cancer with antipsychotic drug use. This review found an increased association of breast cancer with antipsychotic use. These findings support the proposition that there is a real and statistically significant (as well as clinically relevant) increased risk of breast cancer in women who use antipsychotic medications. Stronger warnings about this increased risk are warranted, and regular monitoring of prolactin levels and breast cancer screening should be part of the management plan for these patients.

## CRediT authorship contribution statement

**Steven B Bird:** Writing – review & editing, Writing – original draft, Methodology, Formal analysis, Data curation, Conceptualization.

## Declaration of Competing Interest

The author declares that he has no known competing financial interests or personal relationships that could have appeared to influence the work reported in this paper.

## Data Availability

Data will be made available on request.
